# Agénésie de l’artère pulmonaire gauche

**DOI:** 10.11604/pamj.2016.25.181.10388

**Published:** 2016-11-21

**Authors:** Hicham Naji-Amrani, Yasmina Rhofir

**Affiliations:** 1 Service de Pneumologie, Hôpital Militaire d’Instruction Mohammed V, Rabat, Maroc

**Keywords:** Artère pulmonaire, agénésie, cardiopathie congénitale, Pulmonary artery, agenesis, congenital cardiopathy

## Image en médecine

L’agénésie unilatérale de l’artère pulmonaire est une malformation rare concernant 1% des cardiopathies congénitales et qui peut être source de plusieurs complications. Nous rapportons le cas d’une jeune patiente âgée de 16 ans, sans antécédents pathologiques, présentant une hémoptysie de faible abondance récidivante dans un contexte de conservation de l’état général. L’examen clinique est sans anomalie et la radiographie thoracique de face (A) et de latéral (B) montre un aspect de petit champ pulmonaire gauche, une diminution de la trame vasculaire avec déviation du médiastin à gauche. Devant ce tableau radio-clinique on a évoqué une hypoplasie pulmonaire gauche, séquelles d’infection pulmonaire anciennes ou un corps étranger endobronchique. L’angioscanner thoracique objective l’absence de l’artère pulmonaire gauche (C) avec de nombreuses collatérales systémiques. Le parenchyme pulmonaire gauche est hypoplasique, bien aéré et sans anomalie bronchique (D). À noter que l’aorte descendante est située à droite. L’électrocardiogramme montre un aspect de bloc de branche droit et l’échographie trans-thoracique ne trouve pas d’anomalie cardiaque notamment pas de signe d’hypertension artérielle pulmonaire. La fonction respiratoire à la spiromètrie est normale ainsi que les examens biologiques. L’hémoptysie a été contrôlée par traitement symptomatique et la patiente est sous surveillance médicale.

**Figure 1:**
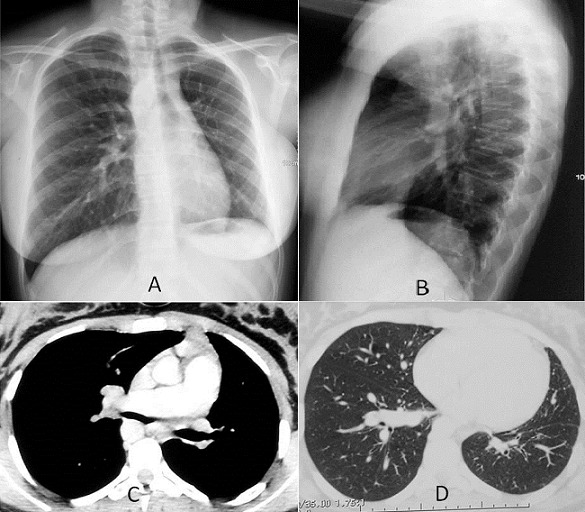
(A, B) radiographie thoracique de face et de profil montrant un petit champ pulmonaire gauche avec déviation médiastinale gauche; C) fenêtre médiastinale de l’angioscanner thoracique objectivant l’absence de l’artère pulmonaire gauche et l’aorte descendante placée à droite; D) fenêtre parenchymateuse du scanner thoracique montrant un poumon gauche hypoplasique mais bien aéré

